# Supplementary material for: Occurrence of Aspergillus and Penicillium Species, Accumulation of Fungal Secondary Metabolites, and qPCR Detection of Potential Aflatoxigenic Aspergillus Species in Chickpea (Cicer arietinum L.) Seeds from Different Farming Systems
Source: Foods. 2025 Jul 25;14(15):2610. doi: 10.3390/foods14152610 (PMC12346705; doi:10.3390/foods14152610)
Supplement: Supplementary file 1 [file foods-14-02610-s001.zip › foods-3726688-supplementary.pdf]

SUPPLEMENTARY MATERIALS

Occurrence of *Aspergillus* and *Penicillium* species, accumulation of fungal secondary metabolites and qPCR detection of potential aflatoxigenic *Aspergillus* species in chickpea (*Cicer arietinum* L.) seeds from different farming systems

Mara Quaglia[a], Francesco Tini[a], Emina Bajrami[a], Erica Quadrini[a], Mariateresa Fedeli[a], Michael Sulyok[b], Giovanni Beccari[a,*], Lorenzo Covarelli[a]

[a]*Department of Agricultural, Food and Environmental Sciences, University of Perugia, Perugia, Italy;*

[b]*Department of Agricultural Sciences, Institute of Bioanalytics and Agro-Metabolomics, BOKU University, Tulln, Austria.*

***Corresponding author:** giovanni.beccari@unipg.it

Table S1. *Aspergillus* spp. isolates used in the phylogenetic analysis and related GenBank accession numbers.

Isolates[1]	Species[2]	Genbank accession number[3]	
		β-tubulin	***Calmodulin***
CBS 557.65[T]	***Aspergillus awamori***	AY820001	AJ964874
CBS 101740[T]	*Aspergillus brasiliensis*	AY820006	AM295175
CBS 566.65[T]	*Aspergillus candidus*	EU014089	EF669550
CBS 111.26[T]	*Aspergillus carbonarius*	AY585532	AJ964873
CBS 115574[T]	*Aspergillus costaricaensis*	AY820014	EU163268
CBS 122712[T]	*Aspergillus eucalypticola*	EU482435	EU482433
NRRL 302[T]	*Aspergillus flavipes*	EU014085	EF669549
CBS 569.65[T]	***Aspergillus flavus***	EF661485	EF661508
CBS 100927	***Aspergillus flavus***	EF203132	EF202063
CBS 133.61[T]	*Aspergillus fumigatus*	EF669791	EF669860
CBS 589.65[T]	*Aspergillus nidulans*	EF652251	EF652339
CBS 569.65[T]	*Aspergillus niger*	AY585536	AJ964872
CBS 115.27[T]	*Aspergillus niveus*	EF669528	EF669573
CBS 108.08[T]	*Aspergillus ochraceous*	EF661322	EF661381
CBS 102.07[T]	*Aspergillus oryzae*	EF661483	EF661506
CBS 100926[T]	***Aspergillus parasiticus***	EF661481	EF661516
CBS 112811[T]	*Aspergillus piperis*	AY20013	EU163267
CBS 756.74[T]	***Aspergillus pseudodeflectus***	EF652331	EF652419
CBS 593.65[T]	*Aspergillus sydowii*	EF652274	EF652362
CBS 103.14[T]	***Aspergillus tamarii***	EF661474	EF661526
CBS 601.65[T]	*Aspergillus terreus*	EF669519	EF669544
NRRL 4875	***Aspergillus tubingensis***	EF661086	EF661151
CBS 134.48[T]	***Aspergillus tubingensis***	AY820007	AJ964876
CBS 113365[T]	*Aspergillus vadensis*	AY585531	EU163269
CBS 139.54[T]	***Aspergillus welwitschiae***	FJ629291	KC480196
CBS 104.07[T]	*Aspergillus wentii*	EF652106	EF652131
CBS 310.38[T]	*Talaromyces flavus*	JX494302	KF741949
CBS 388.87[T]	*Talaromyces marneffei*	JX091389	KF741958
2	*Aspergillus flavus/oryzae*	**PP843161**	**PP843194**
6	*Aspergillus awamori/welwitschiae*	**PP843162**	**PP843195**
33bis	*Aspergillus sydowii*	**PP843178**	**PP843211**
43	*Aspergillus flavus/oryzae*	**PP843163**	**PP843196**
62	*Aspergillus flavus/oryzae*	**PP843164**	**PP843197**
71	*Aspergillus flavus/oryzae*	**PP843165**	**PP843198**
85	*Aspergillus awamori/welwitschiae*	**PP843166**	**PP843199**
89	*Aspergillus flavus/oryzae*	**PP843167**	**PP843200**
93	*Aspergillus pseudodeflectus*	**PP843168**	**PP843201**
95	*Aspergillus awamori/welwitschiae*	**PP843169**	**PP843202**
100	*Aspergillus tubingensis*	**PP843170**	**PP843203**
112	*Aspergillus tubingensis*	**PP843171**	**PP843204**
130	*Aspergillus tubingensis*	**PP843172**	**PP843205**
135	*Aspergillus tubingensis*	**PP843173**	**PP843206**
137	*Aspergillus tamarii*	**PP843174**	**PP843207**
142	*Aspergillus tubingensis*	**PP843175**	**PP843208**
145	*Aspergillus tubingensis*	**PP843176**	**PP843209**
148	*Aspergillus parasiticus*	**PP843177**	**PP843210**

[T]Type strain, as reported by Frisvad et al., 2019; Samson et al., 2014; Varga et al., 2011.

[1]CBS: Culture Collection of the Centraalbureau voor Schimmelcultures, Fungal Biodiversity Centre, Utrecht, The Netherlands; NRRL: National Regional Research Laboratory Mycological Collection, Peoria, IL, USA.

[2]All the species reported in the list has been identified on chickpea or chickpea derivatives (Agarwal et al., 2011; Aguaysol et al., 2013; Ahmad et al., 1993; Ahmad and Singh 1991; Kumar, 2016; Menon, 2017; Mushtaq et al., 2015; Shamsi and Khatum, 2016; Singh, 2014; Sontakke e Hedawoo, 2014; Warude et al., 2016; Youssef et al., 2008), except for the species *A. brasiliensis*, *A. carbonarius*, *A. costaricaensis*, *A. eucalypticola*, *A. piperis*, and *A. vadensis* of the section *Nigri*, that have been used for a better identification of the species obtained from chickpea seed samples analyzed in this research that clustered in this section, according to Varga et al., 2011. Based on the BLAST analysis, the species in bold type are those to which the isolates obtained in the present work showed to belong.

[3]Bold type identified GenBank accession numbers of these isolates obtained in the present work from chickpea

Table S2. *Penicillium* spp. isolates used in the phylogenetic analysis and related GenBank accession numbers.

Isolates[1]	Species	Genbank accession number[3]	
		***β*-tubulin**	**Calmodulin**
CBS 257.29[T]	*Penicillium brevicompactum*	AY674437	AY484813
NRRL 2011	*Penicillium brevicompactum*	DQ645784	AY484817
NRLL 2012	*Penicillium brevicompactum*	DQ645785	AY484818
CBS 300.48[T]	*Penicillium canescens*	JX140946	KJ867009
F727[T]	***Penicillium cellarum***	KM249108	MG714820
F759	***Penicillium cellarum***	MG714865	MG714822
CBS 306.48[T]	***Penicillium chrysogenum***	JF909955	JX996273
CBS 139.45[T]	***Penicillium citrinum***	GU944545	MN969245
CBS 117.64	*Penicillium citrinum*	GU944542	GU944640
CBS 241.85	*Penicillium citrinum*	GU944546	GU944641
CBS 312.48[T]	***Penicillium corylophylum***	JX141042	KP016780
CBS 33079	*Penicillium corylophylum*	GU944519	GU944607
IMI 91917[T]	*Penicillium crustosum*	MN969379	DQ911132
CBS 112082[T]	***Penicillium digitatum***	KJ834447	KU896833
CBS 325.48[T]	*Penicillium expansum*	AY674400	DQ911134
CBS 125543[T]	*Penicillium glabrum*	GU981619	GQ367545
NRRL 35684	*Penicillium glabrum*	EF198564	EF198592
CV 1331	***Penicillium melanoconidium***	JX091545	JX141587
CBS 232.60[T]	*Penicillium olsonii*	AY674445	DQ658165
CBS 219.30[T]	***Penicillium oxalicum***	KF296462	KF296367
CBS 222.28[T]	*Penicillium polonicum*	MN969392	KU896848
CBS 390.48[T]	*Penicillium viridicatum*	MN969406	KU896856
CBS 286.36[T]	*Talaromyces purpurogenus*	JX315639	JX315655
CBS 310.38[T]	*Talaromyces flavus*	JX494302	KF741949
CBS 388.87[T]	*Talaromyces marneffei*	JX091389	KF741958
b10	*Penicillium chrysogenum*	**PP843179**	**PP843212**
b40	*Penicillium chrysogenum*	**PP843180**	**PP843213**
b50	*Penicillium cellarum*	**PP843181**	**PP843214**
b60	*Penicillium chrysogenum*	**PP843182**	**PP843215**
b63	*Penicillium melanoconidium*	**PP843184**	**PP843217**
b71	*Penicillium glabrum*	**PP843185**	**PP843218**
b77	*Penicillium viridicatum*	**PP843186**	**PP843219**
44	*Penicillium canescens*	**PP843183**	**PP843216**
113	*Penicillium crustosum*	**PP843187**	**PP843220**
114	*Penicillium brevicompactum*	**PP843188**	**PP843221**
122	*Penicillium chrysogenum*	**PP843189**	**PP843222**

126	*Penicillium corylophilum*	**PP843190**	**PP843223**
141	*Penicillium expansum*	**PP843191**	**PP843224**
146	*Penicillium expansum*	**PP843192**	**PP843225**
150	*Penicillium olsonii*	**PP843193**	**PP843226**

[T]Type strain, as reported by Houbraken et al., 2020; Frisvad et al., 2019; Samson et al., 2011 and 2014; Varga et al., 2011.
[1]CBS: Culture Collection of the Centraalbureau voor Schimmelcultures, Fungal Biodiversity Centre, Utrecht, The Netherlands; CV: Municipal Museum of Chungking, Sichuwan Province, China; F: Field Museum of Natural History, Botany Department, Chicago, Illinois, USA, IMI: International Mycological Institute, CAB International, United Kingdom; NRRL: National Regional Research Laboratory Mycological Collection, Peoria, IL, USA.
[2]All the species reported in the list has been identified on chickpea or chickpea derivatives (Agarwal et al., 2011; Ahmad et al., 1993; Ahmad e Singh 1991; Menon, 2017; Mushtaq et al., 2015; Patil et al., 2012; Ramirez et al., 2018; Shamsi e Khatum, 2016; Singh, 2014; Sontakke e Hedawoo, 2014; Warude et al., 2016; Youssef et al., 2008). Based on the BLAST analysis, the species in bold are those to which the isolates obtained in the present work showed to belong.
[3]Bold type identified GenBank accession numbers of these isolates obtained in the present work from chickpea seed samples.

Table S3. *Aspergillus* and *Penicillium* species reported on *Cicer arietinum* L. seeds and flour (data updated on 7 June 2025)

Fungal genus	Fungal species	Plant material	Origin	References
Aspergillus	*Aspergillus candidus*	Seed	India	Menon, 2017
	Aspergillus carbonarius	Seed	India	Patil et al., 2012
	Aspergillus flavipes	Seed	India	Menon 2017
	Aspergillus flavus	Seed<br>Flour	Bangladesh<br>India<br>Lybia<br>Pakistan	Dwivedi 1989; Ahmad and Singh, 1991; Javaid et al., 2005; Singh et al., 2005; Dawar et al., 2007; Youssef et al., 2008; Agarwal et al., 2011; Patil et al., 2012; Sontakke and Hedawoo, 2014; Chougule et al., 2015; Mushtaq et al., 2015; Kumar, 2016; Shamsi and Khantum, 2016; Warunde et al., 2016; Menon, 2017; Amule et al., 2019; Shirurkar Deepavali, 2021; Tania et al., 2022
	Aspergillus fumigatus	Seed	Bangladesh<br>India<br>Lybia<br>Pakistan	Javaid et al., 2005; Youssef et al., 2008; Agarwal et al., 2011; Sontakke and Hedawoo, 2014; Kumar, 2016; Shamsi and Khantun, 2016; Menon, 2017; Tania et al., 2022
	Aspergillus nidulans	Seed	Bangladesh	Ahmad and Singh, 1991; Kumar, 2016; Shamsi and Khantun, 2016; Shirurkar Deepavali, 2021; Tania et al., 2022
			India	
	Aspergillus niger (including the sin. *A. ficuum*)	Seed<br>Flour	Australia<br>Bangladesh<br>India<br>Lybia<br>Pakistan	Dwivedi, 1989; Ahmad and Singh, 1991; Nene et al, 1996; Singh et al., 2005; Dawar et al. 2007; Youssef et al., 2008; Agarwal et al., 2011; Patil et al., 2012; Sontakke and Hedawoo, 2014; Mushtaq et al., 2015; Kumar, 2016; Shamsi and Khantun, 2016; Warunde et al., 2016; Menon, 2017; Amule et al., 2019; Shirurkar Deepavali, 2021; Tania et al., 2022
	Aspergillus niveus	Seed	India	Menon, 2017
	Aspergillus ochraceous	Seed	India	Ahmad and Singh, 1991; Sontakke and Hedawoo, 2014; Kumar, 2016; Tania et al., 2022
	Aspergillus oryzae	Seed	India	Menon, 2017; Shirurkar Deepavali, 2021; Tania et al., 2022
	Aspergillus parasiticus	Seed	India<br>Lybia	Youssef et al., 2008; Menon, 2017
	Aspergillus quericinus	Seed	India	Shirurkar Deepavali, 2021
	Aspergillus sydowii	Seed	India	Kumar, 2016
	Aspergillus terreus	Seed	Pakistan	Javaid et al., 2005; Kumar, 2016;
			India	Menon, 2017
	Aspergillus versicolor	Seed	Pakistan	Tania et al., 2022
Penicilllium	*Penicillium canescens*	Seed	Lybia	Youssef et al., 2008
	Penicillium chrysogenum	Seed	Lybia	Youssef et al., 2008
	Penicillium citrinum	Seed	India	Agarwal et al., 2011; Patil et al., 2012
	Penicillium cyclopium	Seed	Lybia	Youssef et al., 2008
	Penicillium digitatum (sin. *P. notatum*)	Seed<br>Flour	India	Singh et al., 2005; Sontakke and Hedawoo, 2014; Mushtaq et al., 2015; Kumar, 2016
	Penicillium expansum	Seed	Lybia	Youssef et al., 2008; Tania et al., 2022
	Penicillium oxalicum	Seed	India	Dwivedi, 1989
	Penicillium viridicatum	Seed	Lybia	Youssef et al., 2008
	Penicillium purpurogenus (currently *Talaromyces purpurogenum)*	Seed	India	Menon, 2017

Table S4. Average number of total fungal colonies or fungal colonies belonging to the *Aspergillus, Penicillium, Cladosporium, Alternaria, Rhizopus* genera or to other genera that developed from each single marketed chickpea seed sample using three different isolation methods [Potato Dextrose Agar with surface disinfection (PDA D), Potato Dextrose Agar without surface disinfection (PDA ND) and deep-freezing blotter (DFB)] or considering all isolation methods together (TOTAL). The genus of each fungal colony was morphologically identified. For each sample, the Total column represents the average (± standard error) of the 15 analyzed plates, each containing 10 seeds, for a total of 150 seeds; the columns for PDA D, PDA ND and DFB each represent the average (± standard error) of five analysed plates, each containing 10 seeds, for a total of 50 seeds per method. In each column, lowercase letters indicate significant difference ($p < 0.05$, Tukey's HSD test) among samples inside each method (PDA D or PDA ND or DFB or TOTAL) while uppercase letters indicate significant difference ($p < 0.05$, Tukey's HSD test) among the three different isolation methods (PDA D, PDA ND or DFB) within each sample.

Sample	Method	Total	*Aspergillus*	*Penicillium*	*Cladosporium*	*Alternaria*	*Rhizopus*	Other
1	**PDA D**	8.4 ± 1.33<br>de B	7.0 ± 1.52<br>de B	0.8 ± 0.58<br>ab A	0.2 ± 0.2<br>a A	0.4 ± 0.4<br>a A	0 ± 0<br>a A	0 ± 0<br>a A
	**PDA ND**	10.2 ± 0.8<br>g B	9.4 ± 0.51<br>g B	0.8 ± 0.37<br>ab A	0 ± 0<br>a A	0 ± 0<br>a A	0 ± 0<br>a A	0 ± 0<br>a A
	**DFB**	2.8 ± 0.2<br>abcd A	1.0 ± 0.63<br>abc A	0.2 ± 0.2<br>a A	0 ± 0<br>a A	0 ± 0<br>a A	0.4 ±0.24<br>a A	1.2 ± 0.73<br>ab A
	**TOTAL**	7.13 ± 0.97<br>def	5.8 ± 1.08<br>f	0.6 ± 023<br>a	0.07 ± 0.07<br>a	0.13 ± 0.13<br>a	0.13 ± 0.09<br>a	0.73 ± 0.27<br>ab
2	**PDA D**	9.6 ± 1.29<br>e B	5.2 ± 0.92<br>d B	0.2 ± 0.2<br>a A	0 ± 0<br>a A	0 ± 0<br>a A	4.2 ± 1.71<br>b B	0 ± 0<br>a A
	**PDA ND**	7.4 ± 0.24<br>defg B	7.0 ± 0<br>efg B	0 ± 0<br>a A	0 ± 0<br>a A	0 ± 0<br>a A	0.4 ± 0.24<br>ab A	0 ± 0<br>a A
	**DFB**	2.2 ± 0.37<br>abc A	0 ± 0<br>a A	0 ± 0<br>a A	0 ± 0<br>a A	0 ± 0<br>a A	0.4 ± 0.4<br>a A	1.8 ± 0.37<br>bc B
	**TOTAL**	6.4 ± 0.93<br>def	4.07 ± 0.84<br>cdef	0.67 ± 0.67<br>a	0 ± 0<br>a	0 ± 0<br>a	1.67 ± 0.73<br>ab	2.27 ± 0.25<br>ab
3	**PDA D**	7.6 ± 1.36<br>de A	5.8 ± 1.11<br>d A	0 ± 0<br>a A	0 ± 0<br>a A	0 ± 0<br>a A	1.2 ± 0.97<br>a A	0.6 ± 0.6<br>a A
	**PDA ND**	7.2 ± 0.49<br>defg A	6.8 ± 0.49<br>efg A	0 ± 0<br>a A	0 ± 0<br>a A	0 ± 0<br>a A	0.4 ± 0.24<br>ab A	0 ± 0<br>a A
	**DFB**	7.4 ± 0.24<br>ef A	5.4 ± 0.93<br>d A	0 ± 0<br>a A	0 ± 0<br>a A	0 ± 0<br>a A	0.2 ± 0.2<br>a A	1.8 ± 0.92<br>bc A
	**TOTAL**	7.4 ± 0.46<br>def	6.0 ± 0.5<br>f	0 ± 0<br>a	0 ± 0<br>a	0 ± 0<br>a	0.6 ± 0.34<br>ab	1.4 ± 0.39<br>ab
4	**PDA D**	7.6 ± 0.51<br>de A	4.8 ± 0.97<br>cd A	2.8 ± 0.86<br>c B	0 ± 0<br>a A	0 ± 0<br>a A	0 ± 0<br>a A	0 ± 0<br>a A
	**PDA ND**	7.8 ± 0.58<br>defg A	7.8 ± 0.58<br>g B	0 ± 0<br>a A	0 ± 0<br>a A	0 ± 0<br>a A	0 ± 0<br>a A	0 ± 0<br>a A
	**DFB**	7.0 ± 0.32<br>ef A	5.6 ± 0.24<br>d AB	0.8 ± 0.37<br>ab A	0 ± 0<br>a A	0 ± 0<br>a A	0 ± 0<br>a A	0.6 ± 0.40<br>ab A
	**TOTAL**	7.47 ± 0.27<br>def	6.07 ± 0.49<br>f	1.2 ± 0.43<br>a	0 ± 0<br>a	0 ± 0<br>a	0 ± 0<br>a	0.2 ± 0.15<br>a
5	**PDA D**	7.0 ± 0<br>cde B	7.0 ± 0<br>de B	0 ± 0<br>a A	0 ± 0<br>a A	0 ± 0<br>a A	0 ± 0<br>a A	0 ± 0<br>a A
	**PDA ND**	2.2 ± 0.8<br>ab A	2.2 ± 0.8<br>abc A	0 ±<br>a A	0 ± 0<br>a A	0 ± 0<br>a A	0 ± 0<br>a A	0 ± 0<br>a A
	**DFB**	5.2 ± 0.58<br>cdef B	5.0 ± 0.71<br>d B	0 ± 0<br>a A	0 ± 0<br>a A	0 ± 0<br>a A	0 ± 0<br>a A	0.2 ± 0.20<br>ab A
	**TOTAL**	4.8 ± 0.61<br>abcde	4.73 ± 0.62<br>def	0 ± 0<br>a	0 ± 0<br>a	0 ± 0<br>a	0 ± 0<br>a	0.07 ± 0.07<br>a
6	**PDA D**	1.6 ± 0.6<br>ab A	1.6 ± 0.6<br>abc AB	0 ± 0<br>a A	0 ± 0<br>a A	0 ± 0<br>a A	0 ± 0<br>a A	0 ± 0<br>a A
	**PDA ND**	2.6 ± 0.24<br>abc A	2.6 ± 0.24<br>abc B	0 ± 0<br>a A	0 ± 0<br>a A	0 ± 0<br>a A	0 ± 0<br>a A	0 ± 0<br>a A

	**DFB**	1.8 ± 0.58<br>abc A	0.8 ± 0.37<br>ab A	0.8 ± 0.2<br>ab B	0 ± 0<br>a A	0 ± 0<br>a A	0.2 ± 0.2<br>a A	0 ± 0<br>a A
	**TOTAL**	2.0 ± 0.29<br>abc	1.67 ± 0.30<br>abcd	0.27 ± 0.19<br>a	0 ± 0<br>a	0 ± 0<br>a	0.07 ± 0.07<br>a	0.07 ± 0<br>a
**7**	**PDA D**	5 ± 1.52<br>bcd A	4.4 ± 1.21<br>cd A	0 ± 0<br>a A	0 ± 0<br>a A	0 ± 0<br>a A	0.6 ± 0.4<br>a A	0 ± 0<br>a A
	**PDA ND**	4.6 ± 0.24<br>bcd A	4.6 ± 0.24<br>cdef A	0 ± 0<br>a A	0 ± 0<br>a A	0 ± 0<br>a A	0 ± 0<br>a A	0 ± 0<br>a A
	**DFB**	4.4 ± 0.40<br>bcde A	4.4 ± 0.4<br>d A	0 ± 0<br>a A	0 ± 0<br>a A	0 ± 0<br>a A	0 ± 0<br>a A	0 ± 0<br>a A
	**TOTAL**	4.67 ± 0.49<br>abcde	4.47 ± 0.40<br>def	0 ± 0<br>a	0 ± 0<br>a	0 ± 0<br>a	0.2 ± 0.14<br>a	0.2 ± 0<br>a
**8**	**PDA D**	10 ± 1.38<br>e B	7.2 ± 0.80<br>de B	0 ± 0<br>a A	0 ± 0<br>a A	0 ± 0<br>a A	2.8 ± 1.71<br>ab A	0 ± 0<br>a A
	**PDA ND**	7.4 ± 0.4<br>defg B	7.4 ± 0.4<br>fg B	0 ± 0<br>a A	0 ± 0<br>a A	0 ± 0<br>a A	0 ± 0<br>a A	0 ± 0<br>a A
	**DFB**	4.2 ± 0.20<br>bcde A	4.2 ± 0.2<br>d A	0 ± 0<br>a A	0 ± 0<br>a A	0 ± 0<br>a A	0 ± 0<br>a A	0 ± 0<br>a A
	**TOTAL**	7.2 ± 0.78<br>def	6.27 ± 0.48<br>f	0 ± 0<br>a	0 ± 0<br>a	0 ± 0<br>a	0.93 ± 0.64<br>ab	0.93 ± 0<br>ab
**9**	**PDA D**	7 ± 1.26<br>cde A	3.8 ± 1.16<br>bcd B	2.4 ± 0.68<br>bc A	0.8 ± 0.8<br>a A	0 ± 0<br>a A	0 ± 0<br>a A	0 ± 0<br>a A
	**PDA ND**	10.4 ± 0.75<br>g AB	7.0 ± 0<br>efg C	3.4 ± 0.74<br>c A	0 ± 0<br>a A	0 ± 0<br>a A	0 ± 0<br>a A	0 ± 0<br>a A
	**DFB**	12.2 ± 1.32<br>g B	0 ± 0<br>a A	7.0 ± 0.0<br>d B	5.2 ± 1.32<br>c B	0 ± 0<br>a A	0 ± 0<br>a A	0 ± 0<br>a A
	**TOTAL**	9.87 ± 0.84<br>f	3.6 ± 0.84<br>bcdef	4.27 ± 0.61<br>b	2 ± 0.77<br>c	0 ± 0<br>a	0 ± 0<br>a	2 ± 0<br>ab
**10**	**PDA D**	5.2 ± 0.58<br>bcd A	0 ± 0<br>a A	5.2 ± 0.58<br>d A	0 ± 0<br>a A	0 ± 0<br>a A	0 ± 0<br>a A	0 ± 0<br>a A
	**PDA ND**	6.0 ± 0.84<br>cdef A	2.2 ± 0.58<br>abc B	3.8 ± 1.36<br>c A	0 ± 0<br>a A	0 ± 0<br>a A	0 ± 0<br>a A	0 ± 0<br>a A
	**DFB**	8.2 ± 0.97<br>f A	0 ± 0<br>a A	5.0 ± 0.71<br>c A	3.2 ± 0.73<br>b B	0 ± 0<br>a A	0 ± 0<br>a A	0 ± 0<br>a A
	**TOTAL**	6.47 ± 0.55<br>def	0.73 ± 0.33<br>ab	4.67 ± 0.53<br>b	1.07 ± 0.46<br>bc	0 ± 0<br>a	0 ± 0<br>a	1.07 ± 0<br>ab
**11**	**PDA D**	2.0 ± 0.32<br>ab A	0.8 ± 0.37<br>ab A	0.8 ± 0.2<br>ab B	0 ± 0<br>a A	0 ± 0<br>a A	0 ± 0<br>a A	0.4 ± 0.24<br>a A
	**PDA ND**	5.2 ± 1.56<br>bcde B	1.8 ± 0.92<br>abc A	0 ± 0<br>a A	0 ± 0<br>a A	0 ± 0<br>a A	3.4 ± 1.47<br>bcde B	0 ± 0<br>a A
	**DFB**	4.8 ± 0.86<br>bcdef AB	3.0 ± 0.84<br>bcd A	0 ± 0<br>a A	0 ± 0<br>a A	1.8 ± 1.36<br>b A	0 ± 0<br>a A	0 ± 0<br>a A
	**TOTAL**	4.0 ± 0.59<br>abcde	1.87 ± 0.47<br>abcd	0.27 ± 0.12<br>a	0 ± 0<br>a	0.6 ± 0.48<br>a	1.13 ± 0.62<br>ab	1.87 ± 0.09<br>ab
**12**	**PDA D**	0 ± 0<br>a A	0 ± 0<br>a A	0 ± 0<br>a A	0 ± 0<br>a A	0 ± 0<br>a A	0 ± 0<br>a A	0 ± 0<br>a A
	**PDA ND**	8.8 ± 1.2<br>efg B	4.2 ± 0.49<br>bcde B	0 ± 0<br>a A	0 ± 0<br>a A	0 ± 0<br>a A	4.6 ± 1.47<br>cde B	0 ± 0<br>a A
	**DFB**	2.4 ± 0.68<br>abc A	1.2 ± 0.37<br>abc A	1.2 ± 0.37<br>ab B	0 ± 0<br>a A	0 ± 0<br>a A	0 ± 0<br>a A	0 ± 0<br>a A
	**TOTAL**	3.73 ± 1.08<br>abcd	1.80 ± 0.51<br>abcd	0.4 ± 0.19<br>a	0 ± 0<br>a	0 ± 0<br>a	1.53 ± 0.74<br>ab	1.53 ± 0<br>ab
**13**	**PDA D**	3.6 ± 0.51<br>abc A	1.4 ± 0.87<br>abc A	0.6 ± 0.6<br>a A	0.8 ± 0.37<br>a B	0.8 ± 0.8<br>a A	0 ± 0<br>a A	0 ± 0<br>a A
	**PDA ND**	7.8 ± 0.48<br>defg B	1.4 ± 0.6<br>ab A	0 ± 0<br>a A	0 ± 0<br>a A	0 ± 0<br>a A	6.4 ± 0.6<br>e B	0 ± 0<br>a A
	**DFB**	6.2 ± 1.69<br>def AB	3.6 ± 1.03<br>bcd A	1.6 ± 0.93<br>ab A	0 ± 0<br>a A	0 ± 0<br>a A	1.0 ± 1.0<br>ab A	0 ± 0<br>a A
	**TOTAL**	5.87 ± 0.73<br>de	2.13 ± 0.53<br>abcd	0.73 ± 0.38<br>a	0.27 ± 0.15<br>ab	0.27 ± 0.27<br>a	2.47 ± 0.83<br>b	3.0 ± 0<br>b
**14**	**PDA D**	0 ± 0<br>a A	0 ± 0<br>a A	0 ± 0<br>a A	0 ± 0<br>a A	0 ± 0<br>a A	0 ± 0<br>a A	0 ± 0<br>a A
	**PDA ND**	0 ± 0<br>a A	0 ± 0<br>a A	0 ± 0<br>a A	0 ± 0<br>a A	0 ± 0<br>a A	0 ± 0<br>a A	0 ± 0<br>a A

	**DFB**	3.0 ± 0.45 abcd B	0 ± 0 a A	0 ± 0 a A	0 ± 0 a A	0 ± 0 a A	0 ± 0 a A	3.0 ± 0.45 c B
	**TOTAL**	1.0 ± 0.40 a	0 ± 0 a	0 ± 0 a	0 ± 0 a	0 ± 0 a	0 ± 0 a	1.0 ± 0.4 ab
**15**	**PDA D**	9.6 ± 0.81 e B	6.4 ± 0.51 de A	0 ± 0 a A	0 ± 0 a A	0 ± 0 a A	0 ± 0 a A	3.2 ± 0.37 b B
	**PDA ND**	9.6 ± 0.81 fg B	6.4 ± 0.51 defg A	0 ± 0 a A	0 ± 0 a A	0 ± 0 a A	0 ± 0 a A	3.2 ± 0.37 b B
	**DFB**	4.0 ± 1.48 bcde A	3.8 ± 1.59 cd A	0 ± 0 a A	0 ± 0 a A	0 ± 0 a A	0 ± 0 a A	0.2 ± 0.20 ab A
	**TOTAL**	7.73 ± 0.91 ef	5.53 ± 0.63 ef	0 ± 0 a	0 ± 0 a	0 ± 0 a	0 ± 0 a	2.2 ± 0.42 ab
**16**	**PDA D**	1.8 ± 0.86 ab A	0.4 ± 0.24 ab A	0 ± 0 a A	0 ± 0 a A	0 ± 0 a A	1.0 ± 1.0 a A	0.4 ± 0.4 a A
	**PDA ND**	10.8 ± 1.16 g B	7.8 ± 1.24 g B	0 ± 0 a A	0 ± 0 a A	0 ± 0 a A	3.0 ± 1.38 abcd A	0 ± 0 a A
	**DFB**	3.2 ± 0.92 abcd A	2.8 ± 0.8 abcd A	0 ± 0 a A	0 ± 0 a A	0 ± 0 a A	0 ± 0 a A	0.4 ± 0.40 ab A
	**TOTAL**	5.26 ± 1.18 bcde	3.67 ± 0.95 bcdef	0 ± 0 a	0 ± 0 a	0 ± 0 a	1.33 ± 0.62 ab	1.6 ± 0.18 ab
**17**	**PDA D**	0.4 ± 0.24 a A	0 ± 0 a A	0.4 ± 0.24 a A	0 ± 0 a A	0 ± 0 a A	0 ± 0 a A	0 ± 0 a A
	**PDA ND**	9.0 ± 0.44 fg C	3.6 ± 0.68 bcd B	0 ± 0 a A	0 ± 0 a A	0 ± 0 a A	5.4 ± 0.68 de B	0 ± 0 a A
	**DFB**	4.4 ± 1.21 bcde B	0 ± 0 a A	2.2 ± 1.32 b A	0 ± 0 a A	0 ± 0 a A	1.0 ± 0.55 ab A	1.2± 0.73 ab A
	**TOTAL**	4.6 ± 1.02 abcde	1.2 ± 0.50 abc	0.87 ± 0.49 a	0 ± 0 a	0 ± 0 a	2.13 ± 0.68 ab	2.53 ± 0.27 ab
**18**	**PDA D**	0.6 ± 0.4 a A	0.2 ± 0.2 a A	0.4 ± 0.24 a A	0 ± 0 a A	0 ± 0 a A	0 ± 0 a A	0 ± 0 a A
	**PDA ND**	6.2 ± 1.11 cdef B	1.8 ± 0.8 abc A	2.4 ± 0.68 bc B	0 ± 0 a A	0 ± 0 a A	2.0 ± 0.94 abc A	0 ± 0 a A
	**DFB**	7.6 ± 0.40 ef B	5.4 ± 0.93 d B	0 ± 0 a A	0 ± 0 a A	0.2 ± 0.2 a A	2.0 ± 0.77 b A	0 ± 0 a A
	**TOTAL**	4.8 ± 0.90 abcde	2.47 ± 0.70 abcde	0.93 ± 0.36 a	0.07 ± 0.07 a	0 ± 0 a	1.33 ± 0.45 ab	1.4 ± 0 ab
**19**	**PDA D**	9.8 ± 0.73 e C	9.8 ± 0.73 e B	0 ± 0 a A	0 ± 0 a A	0 ± 0 a A	0 ± 0 a A	0 ± 0 a A
	**PDA ND**	6.0 ± 1.38 cdef B	2.6 ± 1.29 abc A	0 ± 0 a A	0 ± 0 a A	0 ± 0 a A	3.4 ± 0.75 bcde B	0 ± 0 a A
	**DFB**	1.2 ± 0.37 ab A	0 ± 0 a A	1.2 ± 0.37 ab B	0 ± 0 a A	0 ± 0 a A	0 ± 0 a A	0 ± 0 a A
	**TOTAL**	5.67 ± 1.06 cde	4.13 ± 1.20 cdef	0.4 ± 0.19 a	0 ± 0 a	0 ± 0 a	1.33 ± 0.49 ab	1.13 ± 0 ab
**20**	**PDA D**	0.2 ± 0.2 a A	0 ± 0 a A	0.2 ± 0.2 a A	0 ± 0 a A	0 ± 0 a A	0 ± 0 a A	0 ± 0 a A
	**PDA ND**	4.6 ± 0.68 bcd B	2.4 ± 0.81 abc B	0 ± 0 a A	0 ± 0 a A	0 ± 0 a A	2.2 ± 1.02 abcd B	0 ± 0 a A
	**DFB**	0 ± 0 a A	0 ± 0 a A	0 ± 0 a A	0 ± 0 a A	0 ± 0 a A	0 ± 0 a A	0 ± 0 a A
	**TOTAL**	1.6 ± 0.61 ab	0.8 ± 0.4 ab	0.07 ± 0.07 a	0 ± 0 a	0 ± 0 a	0.73 ± 0.42 ab	0.73 ± 0 ab
